# Identification and evolutionary analysis of the nucleolar proteome of *Giardia lamblia*

**DOI:** 10.1186/s12864-020-6679-9

**Published:** 2020-03-30

**Authors:** Jin-Mei Feng, Chun-Lin Yang, Hai-Feng Tian, Jiang-Xin Wang, Jian-Fan Wen

**Affiliations:** 1 State Key Laboratory of Genetic Resources and Evolution, Kunming Institute of Zoology, Chinese Academy of Sciences, Kunming, 650223 Yunnan Province China; 2 Department of Pathogenic Biology, School of Medicine, Jianghan University, Wuhan, 430056 Hubei Province China; 3 College of Life Sciences and Oceanography, Shenzhen University, Shenzhen, 518006 Guangdong Province China

**Keywords:** *Giardia lamblia*, Protist, Nucleolar proteome, Evolution, Primitiveness, Parasitic reduction

## Abstract

**Background:**

The nucleoli, including their proteomes, of higher eukaryotes have been extensively studied, while few studies about the nucleoli of the lower eukaryotes – protists were reported. *Giardia lamblia*, a protist with the controversy of whether it is an extreme primitive eukaryote or just a highly evolved parasite, might be an interesting object for carrying out the nucleolar proteome study of protists and for further examining the controversy.

**Results:**

Using bioinformatics methods, we reconstructed *G. lamblia* nucleolar proteome (*Gi*NuP) and the common nucleolar proteome of the three representative higher eukaryotes (human, *Arabidopsis*, yeast) (HEBNuP). Comparisons of the two proteomes revealed that: 1) *Gi*NuP is much smaller than HEBNuP, but 78.4% of its proteins have orthologs in the latter; 2) More than 68% of the *Gi*NuP proteins are involved in the “Ribosome related” function, and the others participate in the other functions, and these two groups of proteins are much larger and much smaller than those in HEBNuP, respectively; 3) Both *Gi*NuP and HEBNuP have their own specific proteins, but HEBNuP has a much higher proportion of such proteins to participate in more categories of nucleolar functions.

**Conclusion:**

For the first time the nucleolar proteome of a protist - *Giardia* was reconstructed. The results of comparison of it with the common proteome of three representative higher eukaryotes -- HEBNuP indicated that the simplicity of *Gi*NuP is most probably a reflection of primitiveness but not just parasitic reduction of *Giardia*, and simultaneously revealed some interesting evolutionary phenomena about the nucleolus and even the eukaryotic cell, compositionally and functionally.

## Background

Nucleolus, the most prominent sub-nuclear compartment in the interphase nucleus of eukaryotic cells, is a ribosome factory, where most of the ribosome biogenesis events take place, such as ribosome RNA (rRNA) synthesis, processing, and subsequent assembly of ribosome subunits. Accumulated studies in the past decades have shown that this organelle is also involved in many other cellular processes, such as DNA repair, regulation of mitosis, stress response, biogenesis of multiple ribonucleoprotein particles, cancer, protein quality control [1–6]. Although the multiple functions of the nucleolus have been recognized gradually, when and how they arose in the evolution of eukaryotic cells is still elusive.

The functions of the nucleolus have been studied extensively and deeply in model organisms from the three so-called higher eukaryote groups (animals, plants, and fungi) such as human, *Arabidopsis*, and budding yeast, and the nucleolar proteomes of the three model eukaryotes have already been identified [7–9]. Continuous high-throughput and individual case studies in these higher eukaryotes have identified many nucleolar proteins, indicating potential multiple functions of their nucleoli [10]. However, few studies of nucleoli were carried out in the so-called lower eukaryotes, protists, much less the study of their nucleolar proteomes. It is known that protists occupy pivotal positions in the evolution of eukaryotes because they are the link between prokaryotes and multicellular/higher eukaryotes, and therefore, studies on their nucleoli will be valuable for understanding the origin and evolution of the nucleolus and even the eukaryotic cells.

*Giardia lamblia* is an intestinal protozoan parasite responsible for widespread diarrheal disease in humans and animals worldwide [11]. Besides medical importance, its significance in the study of eukaryotic evolution was first proposed in 1980s but has been debated for many years. It was once thought to be the most primitive extant eukaryote because of having many so-called primitive traits: lack of some eukaryotic typical cellular structures such as mitochondrion [12] and nucleolus [13, 14], and early branching position on some phylogenetic trees [15–18]. However, the later discoveries of mitochondrion-derived organelle -- mitosome [19] and nucleolus [20] in its cells, and the non-early branching positions on some other phylogenetic trees [21, 22] tend to refute the primitivity of *Giardia* but prove that it is just a highly evolved parasite with many parasitic reductions [23, 24]. But on the other hand, some authors found that some simple/primitive traits of *Giardia* cannot be attributed to its parasitic reduction, and still persisted in that *Giardia* is one of the most primitive extant eukaryotes, and emphasized that it is of significance to the study of the evolution of the eukaryotic cell [25–28]. Therefore, the study of the nucleolar proteome of *G. lamblia* may be useful either to the re-examining of the debate above or to the understanding of the evolution of the nucleolus and the eukaryotic cell.

However, high quality isolation of nucleoli from nuclei is always a challenge even for higher eukaryotic cells using the already-existing experimental techniques, and it is much more difficult to *G. lamblia* because of the smallest size of its nucleolus and probably other reasons such as its fragility. Accordingly, it is almost impossible to use mass spectrometry, the best efficient method for proteome studies, to identify nucleolar proteins of *G. lamblia* so far. Fortunately, the nucleolar proteomes and genome databases of three higher eukaryotic representatives of animals, plants, and fungi mentioned above are available, and the completely sequenced genome of *G. lamblia* has also been determined and reported. Therefore, here we used a series of bioinformatics tools to identify nucleolar protein genes of *G. lamblia* and reconstruct the nucleolar proteome (*Gi*NuP) and also to reconstruct the ‘Higher Eukaryote Basic Nucleolar Proteome (HEBNuP)’, then a comprehensively comparative proteomics analysis between the *Gi*NuP and the HEBNuP were performed, and thus some significant implications for the evolution of nucleolar protein components and functions and for the evolutionary position of *Giardia* were obtained.

## Results

### Reconstruction of the giardial nucleolar proteome (*Gi*NuP)

To obtain a relatively complete nucleolar proteome of *G. lamblia*, we have used two independent methods to bioinformatically identify putative nucleolar proteins in the genome of this protist: homology search based on the known nucleolar proteins of the three higher eukaryote representatives and de novo prediction by analyzing protein sequence features. For homology search, 38 candidate *Giardia* orthologs were obtained when blasting with 209 yeast nucleolar proteins as queries. Analogously, 57 and 189 candidate orthologs were obtained when blasting with 217 *A. thaliana* and 4057 human nucleolar proteins as queries, respectively. All the *Giardia* nucleolar proteins orthologous to those of *H. sapiens, A. thaliana,* and *S. cerevisiae* were collected together. After discarding the redundant ones, 237 *Giardia* nucleolar protein candidates were obtained. Subsequent domain analyses of these obtained protein sequences by using PFAM online service showed that 216 ones possess characteristic domains of various nucleolar proteins. They were further confirmed to be nucleolar proteins by Blast searching against the non-redundant (nr) protein database in National Center for Biotechnology Information (NCBI). Finally, 216 orthologs to the nucleolar proteins of the three representative eukaryotes were identified in the *G. lamblia* genome database by the homology search approach (Supplementary Table S1).

Since all the available nucleolar proteomes of the three higher eukaryotes each possess their own specific proteins that do not have any homologs in the other two proteomes, it is reasonable to image that *G. lamblia*, though much more ancient, also has its own specific nucleolar proteins, which are not present in other species. Therefore, to identify such putative *Giardia* specific nucleolar proteins, we investigated all the *Giardia* proteins in the genome database to identify those ones that would be predicted to localize to the nucleolus from all the nuclear proteins. First, we got 172 *Giardia* nuclear proteins by predicting to have nuclear location signal. We also used ‘nucleus/nuclear’ or “nucleolus/nucleolar” as key words to screen the *G. lamblia* genome database, and obtained 25 annotated nuclear/nucleolar proteins. Then all the 197 (172 + 25) nuclear proteins were further subjected to the protein sub-localization prediction, and 55 of them were predicted to be most likely localized to the nucleolus.

Altogether, finally 255 (216 + 39) nucleolar proteins were identified in the *G. lamblia* genome database after discarding the redundant ones, which includes 216 orthologs to the nucleolar proteins of the three representative eukaryotes and 39 *Giardia*-specific nucleolar proteins (Supplementary Table S1). Based on the reported RNA-Seq data of *G. lamblia* [29], 246 of the 255 identified nucleolar proteins in the *G. lamblia* genome database were predicted from the transcriptome and genome annotation confirmed, indicating that most of the identified nucleolar proteins in the *G. lamblia* genome was transcribed in trophozoites grown in vitro.

Thus, we have reconstructed a putative nucleolar proteome of *G. lamblia* (*Gi*NuP), which contains 255 individual nucleolar proteins.

### Reconstruction of the ‘Higher Eukaryote Basic Nucleolar Proteome (HEBNuP)’

**Fig. 1:**
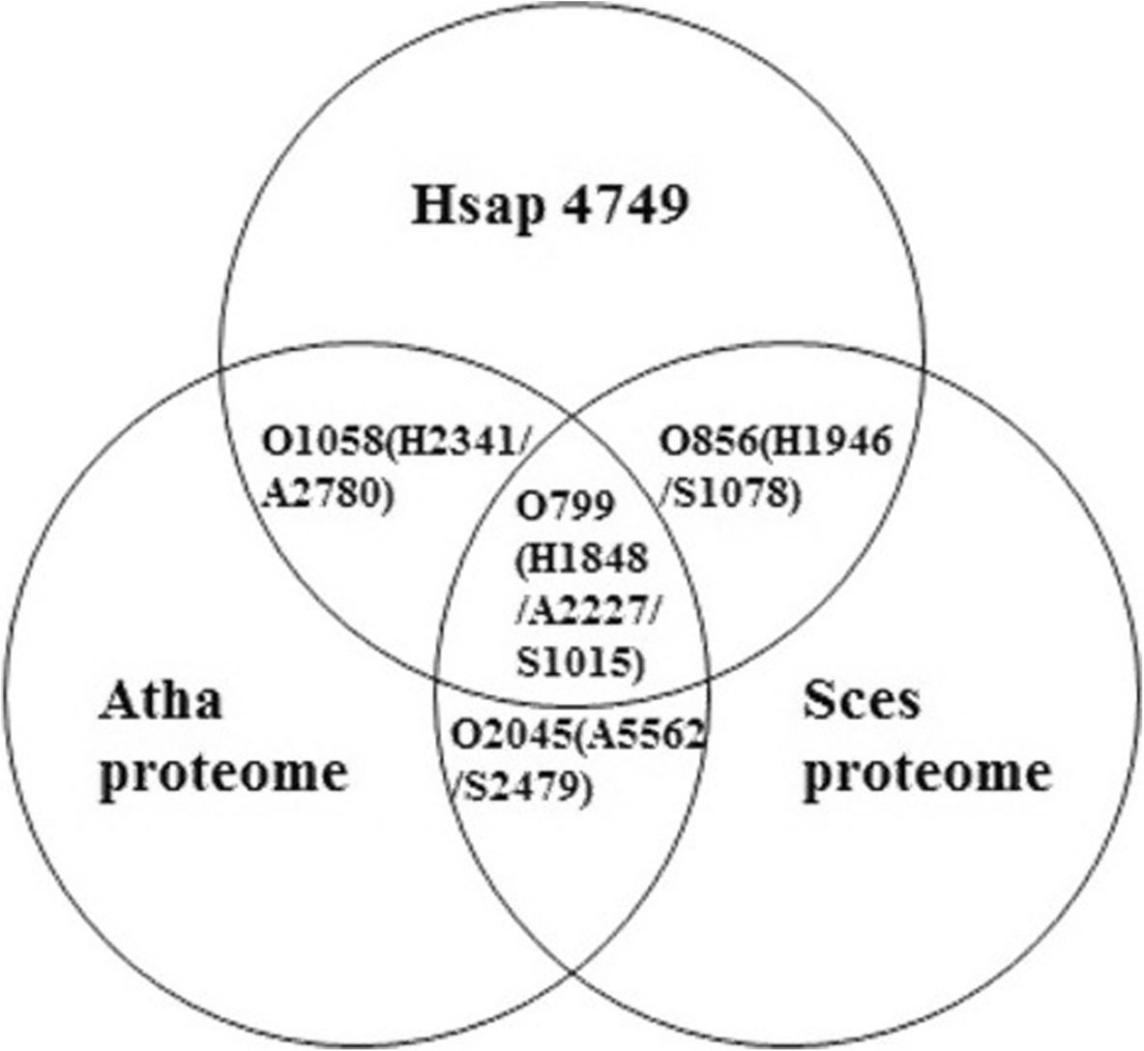
Orthologous relationships of nucleolar proteomes among Human (Hsap, H) and *Arabidopsis* (Atha, A), Yeast (Sces, S)

To compare the *Gi*NuP with the nucleolar proteomes of the three representatives of higher eukaryotes, we investigated the orthologous relationships between either two or among all the three higher eukaryotes by identifying the nucleolar proteins that are present in all the three genomes. Because of the relatively far less protein numbers in both the nucleolar proteomes of *Arabidopsis* and budding yeast, to avoid the possible incompleteness of them, we collected all the ortholog groups with the presence of human nucleolar proteins. This investigation revealed the following orthologous relationships: 1) there are 1058 orthologous groups between human nucleolar proteome and *Arabidopsis* whole proteome, containing 2341 human nucleolar proteins and 2780 *Arabidopsis* proteins, respectively; 2) there are 856 orthologous groups between human nucleolar proteome and budding yeast whole proteome, containing 1946 human nucleolar proteins and 1078 yeast proteins, respectively; 3) there are 799 orthologous groups among human nucleolar proteome, the whole proteome of *Arabidopsis*, and budding yeast proteome, containing 1848 human nucleolar proteins, 2227 *Arabidopsis* proteins, and 1015yeast proteins, respectively (Fig. 1 and Supplementary Table S2). As a whole, we called these 799 orthologous groups as ‘Higher Eukaryote Basic Nucleolar Proteome (HEBNuP)’.

### The functional inventories of the proteins in the HEBNuP and the *Gi*NuP

**Fig. 2:**
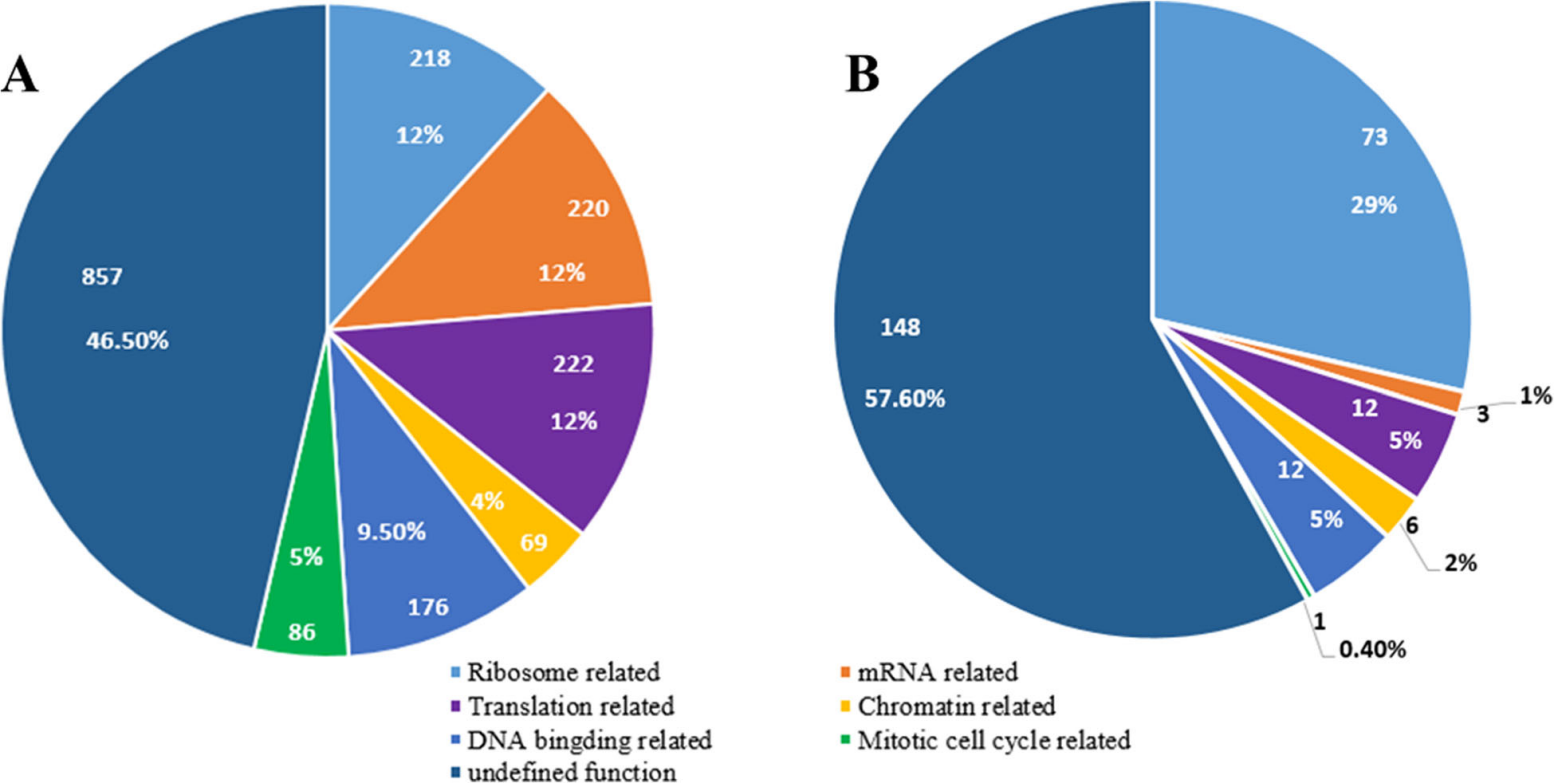
The functional inventories of nucleolar proteins in HEBNuP (**a**) and *Gi*NuP (**b**)

The results of functional inventory of the 1848 human nucleolar proteins in the HEBNuP is as follows (Fig. 2a): 1) 218 (12%) belong to the “Ribosome related” class; 2) 220 (12%) belong to the “mRNA related” class; 3) 222 (12%) belong to the “Translation related” class; 4) 176 (9.5%) belong to the “DNA binding” proteins; 5) 69 (4%) belong to the “Chromatin related” class; 6) 86 (5%) belong to the “Mitotic cell cycle related” class; 7) 857 (46.5%) belong to none of the six classes, and thus we classify them as “undefined function” class.

The results of functional inventory of the 255 proteins in the *Gi*NuP is as follows (Fig. 2b): 1) 73 (29%) proteins are classified among the “Ribosome related” proteins; 2) three (1%) belong to the “mRNA related” class; 3) 12 (5%) belong to the “Translation related” class; 4) 12 (5%) belong to the “DNA binding related” class; 5) six (2%) belong to the “Chromatin related” class; 6) one (0.4%) belong to the “Mitotic cell cycle related” class; 7) 148 (57.6%) belong to the “undefined function” class.

### Comparative analysis between the *Gi*NuP and the HEBNuP

To explore the evolution of nucleolus, we compared the *Gi*NuP and the HEBNuP in terms of protein homology and function. From the above results, we know that the HEBNuP consists of 799 orthologous groups, which contains 1848 individual human nucleolar proteins -- the HEBNuP-*Hu* protein dataset, and that the *Gi*NuP dataset contains 255 orthologous groups and *Giardia* nucleolar proteins. Since the nucleolar proteome of human seems to be the most complete one among those of the three higher eukaryotes, thus the nucleolar protein groups in HEBNuP-*Hu* protein dataset were used as representatives of HEBNuP to compare with those in *Gi*NuP in the following analysis.

Comparison of the *Gi*NuP with the HEBNuP in terms of protein homology shows that: 1) 200 orthologous groups (containing 200 individual *Giardia* nucleolar proteins) are shared by *Gi*NuP and HEBNuP, which make up the HEBNuP-*Gi*NuP-shared dataset, indicating that 78.4% (200 out of 255) of the *Giardia* nucleolar protein orthologous groups (also the individual proteins) all have their orthologs in the HEBNuP, but these orthologs only occupy 25.0% of the orthologous protein groups of the HEBNuP (and the *Giardia* nucleolar proteins only occupy 13.8% of the individual human nucleolar proteins in the HEBNuP and HEBNuP-Hu), which means that the majority of *Giardia* nucleolar proteins belong to the common/basic nucleolar proteins of the higher eukaryotes, and in higher eukaryotes the common/basic nucleolar proteins are much more than in *Giardia*; 2) 55 *Giardia* nucleolar orthologous groups (containing 55 individual *Giardia* nucleolar proteins) are specific to *Gi*NuP, which make up the dataset we call *Gi*NuP-specific datase; 599 orthologous groups (containing 1253 individual human nucleolar proteins) in HEBNuP are specific to HEBNuP, which make up the dataset we call HEBNuP-specific dataset.

**Fig. 3:**
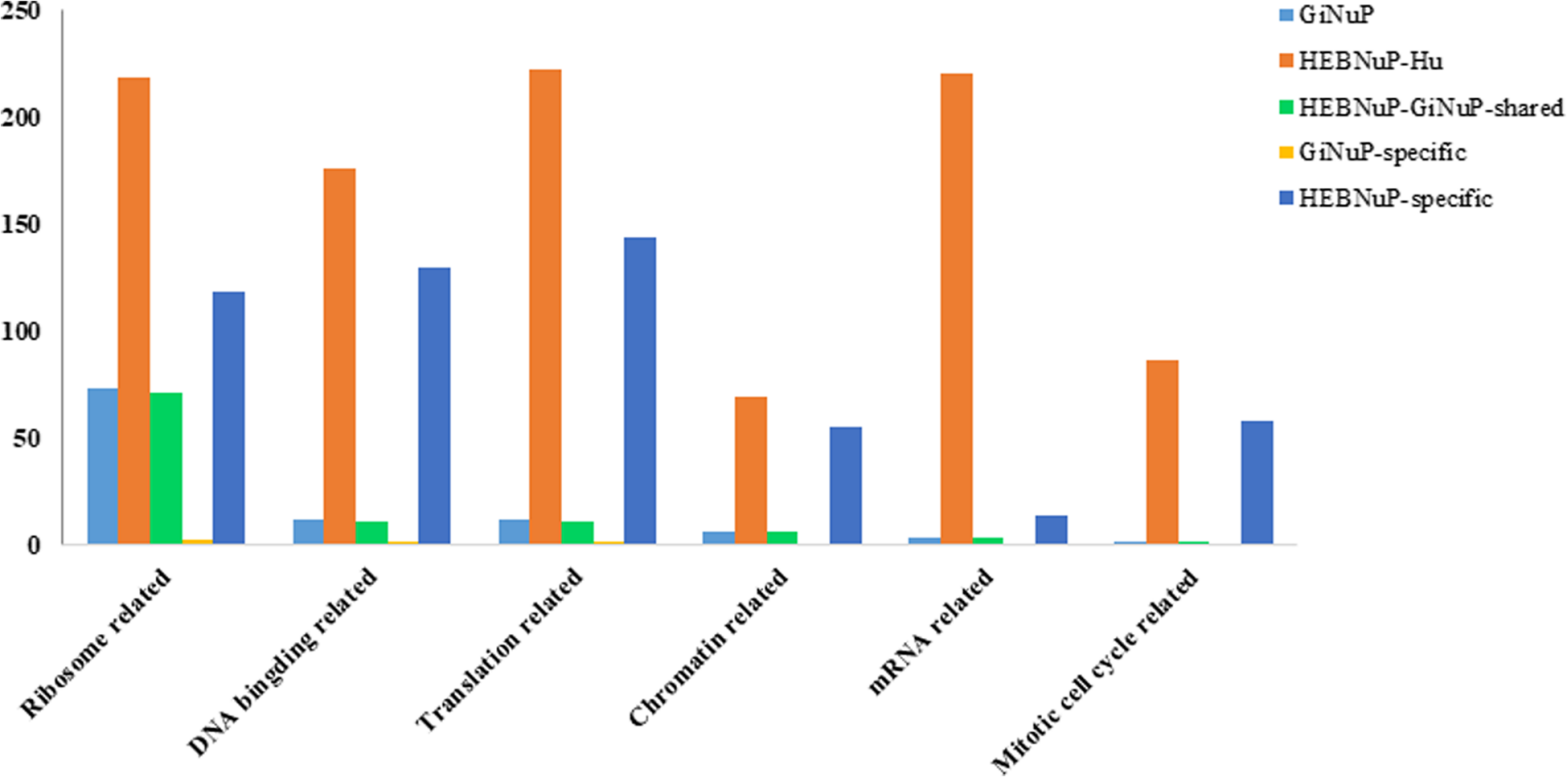
Functional distribution of nucleolar proteins in the five datasets. The five different colors refer to the five datasets, respectively; Horizontal axis, six main and well-known nucleolar functional classes; Vertical axis, Number of proteins

**Fig. 4:**
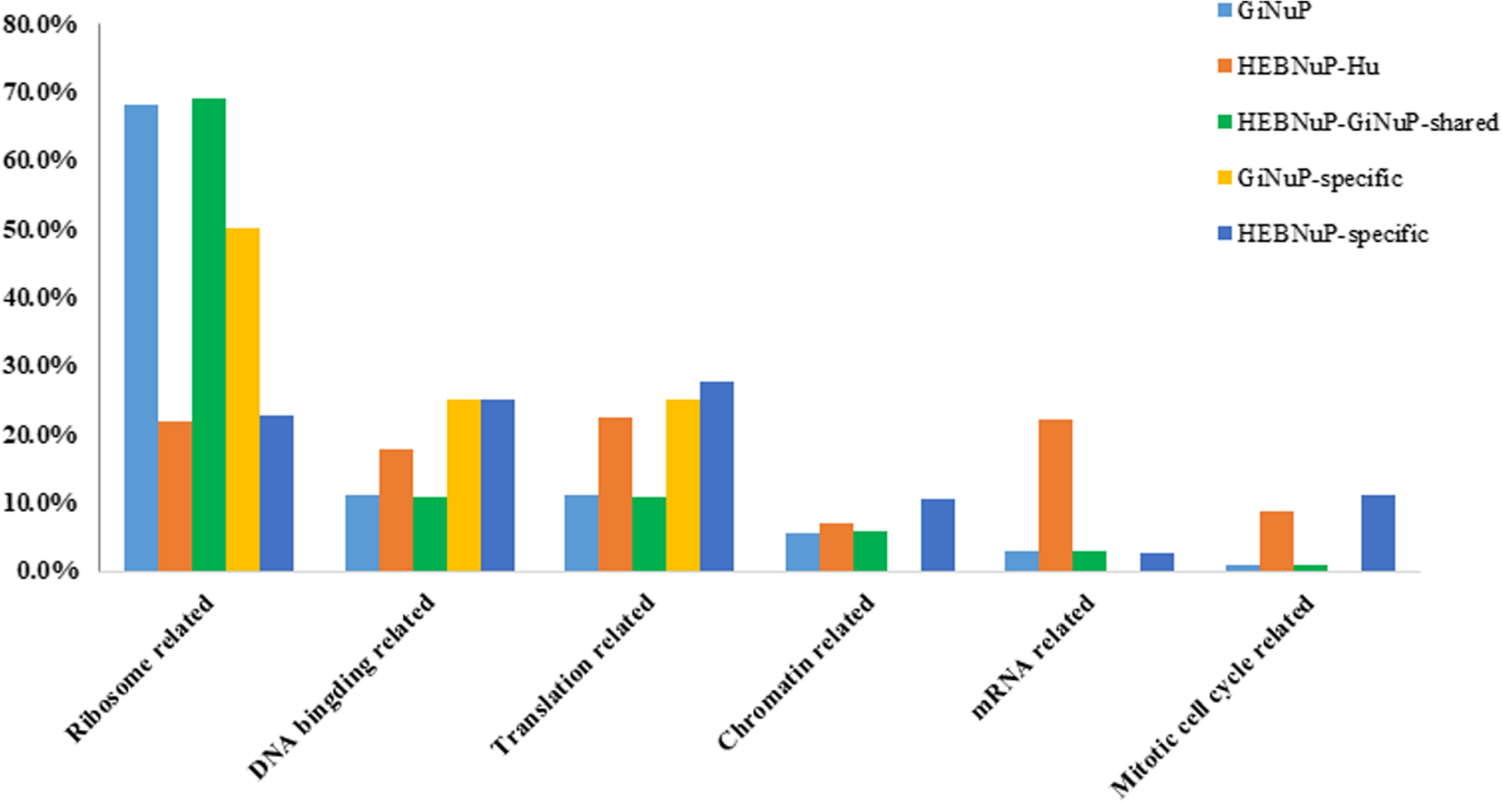
Comparisons of the proportions of the proteins in each nucleolar functional class of the five datasets. The five different colors refer to the five datasets, respectively; Horizontal axis, six main and well-known nucleolar functional classes; Vertical axis, Ratio

The functional distributions of the nucleolar orthologous protein groups in the five datasets mentioned above are shown in Fig. 3, and the proportions of the annotated proteins for each nucleolar functional class are shown in Fig. 4. Functional distribution comparison of the proteins in the *Gi*NuP with those in the HEBNuP shows that: 1) 68.2% of the annotated proteins in the *Gi*NuP dataset and 68.9% in the HEBNuP-*Gi*NuP-shared dataset are involved in the “Ribosome related” function, respectively, implying that the majority of the annotated *Giardia*’s nucleolar proteins participate in the “Ribosome related” function, and that these proteins still perform this function in higher eukaryotes; the other about 31% of the annotated proteins in these two datasets are involved in the other five functions, respectively, implying that besides the major “Ribosome related” function, the other five nucleolar functions also exist in *Giardia*’s nucleolus, though with a very few proteins to perform them, and that these few proteins still perform the five functions in higher eukaryotes. 2) Half (50%) of the annotated proteins in *Gi*NuP-specific dataset are classified into the “Ribosome related” functional class, 25% are classified into the “DNA binding related” functional class, and the other 25% are classified into the “Translation related” functional class, and none are classified into the other three functional classes; 22.7, 25, 27.7, 10.6, 2.7, and 11.2% of the annotated proteins in HEBNuP-specific dataset are classified into the “Ribosome related”, “DNA binding related”, “Translation related”, “Chromatin related”, “mRNA related”, and “Mitotic cell cycle related” functional classes, respectively, which means that the basic “Ribosome related” function of nucleolus also needs lineage- and even species-specific protein components to perform it in a certain lineage or species, and so do the other five nucleolar functions; and that such specific proteins, especially those for the other five functions, continuously increased in the evolution of eukaryotes. Besides, obviously, for both the *Gi*NuP and the *Gi*NuP-specific datasets, the proportions of annotated proteins involved in the other five functional classes all are much fewer than those involved in the “Ribosome related” function, while for the HEBNuP-*Hu* dataset and the HEBNuP-specific dataset, the proportions of nucleolar proteins involved in the other five functions increase much more substantially, compared with those involved in the “Ribosome related” function. This implies that the “Ribosome related” function should arise and consummate earlier than the other five functions, and the other five ones became more and more consummate and complicated latter, especially in the evolution of higher eukaryotes.

## Discussion

The nucleolus of *G. lamblia* seems to be the smallest one described so far [30] and atypical when compared with those of higher eukaryotes [20], and they are very difficult to isolate in high quality for mass spectrometry, thus, here we tried to use bioinformatics methods to identify its proteome based on its genome database and the already-existing nucleolar proteome databases of three representative eukaryotes, human, *Arabidopsis*, and yeast. In order to exhaustively identify the putative nucleolar proteins in *Giardia*, the nucleolar proteins homologous to those of higher eukaryotes and *Giardia*-specific nucleolar proteins were both identified by our combined computational approach. Thus we reconstructed the first nucleolar proteome of unicellular eukaryotes (protists) -- *Giardia*’s nucleolar proteome, *Gi*NuP. Of course, this *Gi*NuP might still be incomplete, because there might still exist some novel nucleolus proteins in *G. lamblia*, which do not bear similarity to other proteins and also do not possess the features of nucleolar protein sequences, might have not been found in this work. If they really exist, they will be able to be identified by using the mass spectrometry after the experimental techniques of isolating the nucleoli from *G. lamblia* cells are developed in the next future. But they might not be many, if any, and their absence in our reconstructed *Gi*NuP may not significantly affect the analyses of the results and the conclusions we reached in the present work.

When comparing with any one of the nucleolar proteomes of human, *A. thaliana*, and yeast [7–9], the *Gi*NuP was found to contain far fewer nucleolar proteins. Thus, in terms of protein number, the nucleolar protein components of *G. lamblia* are much simpler than those of higher eukaryotes. However, since many species-specific nucleolar proteins have been found in the nucleolar proteomes of human, *A. thaliana*, and yeast [7–9], and also in *Giardia* (please see those we identified above), to reasonably compare the component and the function of nucleolar proteins between *Gi*NuP and the nucleolar proteomes of typical eukaryotes, here we reconstructed the HEBNuP, which consists of the nucleolar protein orthologous groups shared by the proteomes of the three representative eukaryotes and thus to a certain degree can represent the common/basic protein components of the nucleolus of higher eukaryotes, and then compared it with the *Gi*NuP in two aspects -- orthologous group and functional category. Compared with that of human, which was obtained by using multiple mass spectrometry to analyze highly purified preparations of human nucleoli from different cell lines, the nucleolar proteomes of *Arabidopsis* and yeast are remarkably smaller and thus might have been underestimated, probably due to the less sensitive mass spectrometric techniques used and the dynamic behavior of nucleolar proteins [8, 9, 31]. Thus in the present work, for *Arabidopsis* and yeast, we used their putative whole proteome (downloaded from the genome database) instead of just their nucleolar proteomes in the reconstruction of HEBNuP. Comparisons of protein components between the *Gi*NuP and the HEBNuP revealed that the majority of *Giardia* nucleolar proteins belong to this common/basic nucleolar proteins of higher eukaryotes, but the individual protein number (and also the orthogous group number) of these *Giardia* nucleolar proteins is far fewer than those in the higher eukaryotes, which suggests that *Giardia*’s simplified nucleolus is most probably a reflection of its primitiveness rather than its parasitic reduction. Because (1) in general, the common/basic nucleolar proteins should emerge earlier than other proteins in the evolution of the nucleolus (and also of the eukaryote), thus our findings that *Gi*NuP is mainly composed of the common/basic nucleolar proteins (namely, the proportion of the other proteins in *Gi*NuP is much lower than that in HEBNuP), and that the main and basic function of nucleolus -- “Ribosome related” function is the major function of the *Gi*NuP, both imply that *Giardia*’s nucleolus is a very primitive one; (2) the parasitic reduction should not be necessary to occur on the common/basic nucleolar proteins which take part in the basic nucleolar function in all eukaryotes but are not directly related to parasitic life-style, and the much smaller number of the common/basic nucleolar proteins in *Giardia* must be due to the primitive status of nucleolus of this organism, and later more and more proteins were recruited into the nucleolus as common/basic nucleolar proteins during eukaryotic evolution after the divergence of *Giardia* from the eukaryote trunk (our data shows that the common/basic nucleolar proteins have increased about 300% from *Gi*NuP to the HEBNuP), on the contrary, it is much less likely that *Giardia* lost so much of the common/basic nucleolar proteins of the eukaryotic essential structure due to parasitism. Actually, our previous studies have also revealed that *Giardia*’s unusual and simple 5S rRNA system is most likely a reflection of its primitiveness but not be due to parasitic degeneration [27], and that *Giardia* possesses 89 orthologs to the 129 conserved common ribosomal biogenesis proteins of higher eukaryotes, which can carry out all the steps of ribosome biogenesis, also indicated that the ribosome biogenesis system of *Giardia* is similar to that of higher eukaryotes but just simpler [32]. Moreover, it was reported that compared with its counterparts in higher eukaryotes, the nucleolar organizer regions (NORs) of *Giardia* gather much less copies of much shorter rDNA repeat units and participate in the formation of the structurally simpler nucleolus of this organism [33]. Therefore, the nucleolus of *G. lamblia* is simpler than those of higher eukaryotes in structure, composition, and function, and such a simplified nucleolus in *G. lamblia* is most probably due to its primitiveness but not secondary parasitic reduction. Our recent work on *Giardia*’s glycerophospholipid (GPL) biosynthesis pathways revealed that these pathways of it are evolutionarily primitive, but with many secondary parasitic adaptation ‘patches’ including gene loss, rapid evolution, and horizontal gene transfer, which implies *Giardia* might be a mosaic of ‘primary primitivity’ and ‘secondary parasitic adaptability’ [28]. This is also consistent with the present work.

Based on the above understanding that *Giardia*’s nucleolus is a primitive one, our results of comparison of the *Gi*NuP with the HEBNuP thus can reveal some interesting evolutionary phenomena. First, the two observations that the majority of *Giardia* nucleolar proteins have orthologs to the common/basic nucleolar proteins of higher eukaryotes (HEBNuP) but occupy a very small proportion of the latter, and that the majority of the *Giardia*’s nucleolar proteins participate in the “Ribosome related” function both may imply that the “Ribosome related” function, as the major/basic function of the nucleolus, must have arisen earlier than the other nucleolar functions, and that this major/basic function became more and more consummate and complicated in the evolution of eukaryotes by increasing more and more functional protein components. Second, there are some proteins in *Gi*NuP (though very few compared to those of higher eukaryotes) involved in the other five nucleolar functions may mean that besides the major “Ribosome related” function, the other five nucleolar functions also have arisen in *Giardia*, though with a very few proteins to perform them, and these functions also became more and more consummate and complicated in the evolution of eukaryotes, especially in the evolutionary process from primitive unicellular protists to higher multicellular eukaryotes. Third, that in either *Giardia* or the higher eukaryotes, either the major “Ribosome related” function or the other five functions, all contain some (quite a proportion in higher eukaryotes) species- and linage-specific proteins, and that such specific proteins, especially those for the other five functions, increased remarkably in higher eukaryotes, both may mean that in all eukaryotic species and lineages, specific protein components are also necessary to evolve to participate in the performance of all the common functions of nucleolus. This might be a very interesting evolutionary biology finding, which probably implies that the evolution from lower to higher organisms, especially in the divergence of species and lineages, does not simply mean the increase of common components on the basis of the relatively lower organisms but the evolutionary emergence of species- and lineage-specific components for a cellular structure or a function so as to became more efficient and consummate in a certain species and lineage.

## Conclusions

To sum up, in the present work for the first time the nucleolar proteome of a lower eukaryote (protist) – *Giardia* (*Gi*NuP) was reconstructed. The results of comparison of it with the common proteome of three representative higher eukaryotes -- HEBNuP indicated that the relatively simple *Gi*NuP is most probably a reflection of the primitiveness but not the parasitic reduction of *Giardia*, and revealed some interesting evolutionary phenomena about the nucleolus and even the eukaryotic cell, compositionally and functionally.

## Methods

### Data collection

The International Protein Index (IPI) IDs of 4749 available *Homo sapiens* nucleolar proteins and their corresponding sequences were retrieved from the Nucleolar Proteome Database NOPdb3.0 [7], and the non-redundant 4057 IDs and sequences were used in this study. The whole human genome data was downloaded from Ensembl. The non-redundant IDs and sequences of 217 available *A. thaliana* nucleolar proteins were downloaded from the Arabidopsis Information Resource [34, 35] and the Arabidopsis nucleolar protein database (AtNoPDB) [36]. The non-redundant IDs and sequences of 209 available *S. cerevisiae* nucleolar proteins were downloaded from the Saccharomyces Genome Database [37–39] and the Comprehensive Yeast Genome Database [40]. The IDs and sequences of the nucleolar proteins of *Homo sapiens*, *A. thaliana*, and *S. cerevisiae* used were collected in Supplementary Table S3. The *G. lamblia* genome data was downloaded from the GiardiaDB (http://giardiadb.org/giardiadb/) [11]. The Gene Ontology (GO) functional annotations of human proteins were downloaded from the Gene Ontology (http://www.geneontology.org/).

### Identification of *Giardia* nucleolar proteins and reconstruction of *G. lamblia* nucleolar proteome (*Gi*NuP)

We used the Best Reciprocal Hit (BRH) method to identify nucleolar protein orthologs in *G. lamblia* genome. Briefly, the nucleolar protein sequences from human, *Arabidopsis,* and budding yeast were used as queries to BLASTP search against *G. lamblia* genome (E-value ≤0.001, coverage ≥25%, and identity ≥25%). The obtained hit protein sequences were collected and used as queries to BLASTP search against genomes of human, *Arabidopsis* and budding yeast following the same standards, respectively. Reciprocal best hits between *G. lamblia* and either of human, *Arabidopsis* and budding yeast were established, and those *Giardia* proteins that have reciprocal hit in either of these three reference genomes were considered as candidate nucleolar proteins in *G. lamblia*. Then, the obtained candidate protein sequences were assessed by domain analysis by using PFAM online service [41], and those ones that contain known nucleolar protein domains were considered as putative nucleolar proteins. Further validation of these putative nucleolar proteins was performed by using them as queries to BLASTP search against GenBank non-redundant (nr) protein database to investigate the annotations of their identified homologs in nr database.

For the nucleolar proteins specific to *G. lamblia,* we identified them by a combined computational approach. First, two approaches were used to screen for nuclear proteins in the *G. lamblia* genome data: 1) Using “nucleus/nuclear” or “nucleolus/nucleolar” as key words to search against the genome database to collect all the related annotated proteins; 2) Using PredictNLS program (https://rostlab.org/owiki/index.php/PredictNLS) and Psort II program (http://psort.hgc.jp) [42] to predict the nuclear location signal (NLS) in all the proteins in the *G. lamblia* genome data and collecting the proteins with NLS. Putting the results of 1) and 2) together, we obtained all the nuclear proteins in the *G. lamblia* genome data. Then, the ProLoc prediction program [43], the SubNucPred program [44], and the LOCTREE3 program [45] were used to predict the subnuclear localizations of them. After comparing the algorithms and corresponding prediction results of the three protein subnuclear localization programs, those ones that were predicted to be localized to the nucleolus by the ProLoc prediction program were considered as nucleolar protein candidates. Finally, after removing those ones overlapping with those identified by BRH above, *Giardia*-specific nucleolar proteins were obtained (Supplementary Table S4).

**Fig. 5:**
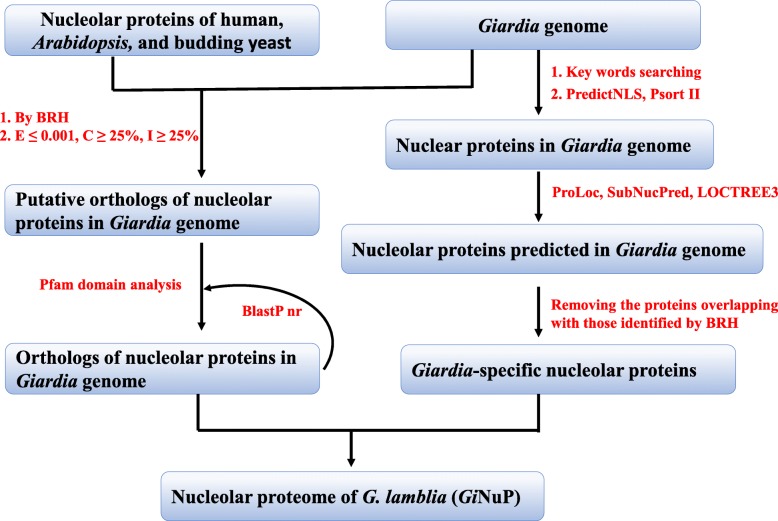
The flow chart of the computational identification of *G. lamblia* nucleolar proteins and the reconstruction of *G. lamblia* nucleolar proteome (*Gi*NuP). E: E-value, C: coverage value, I: Identity value. BRH: Best Reciprocal Hit

Combining the orthologs identified by BRH and the *Giardia*-specific nucleolar proteins ones, we obtained the nucleolar proteins and genes in *G. lamblia* genome data, and put them together and reconstructed *G. lamblia* Nucleolar Proteome (*Gi*NuP). Also, the protein coding genes in *Gi*NuP were predicted from the reported transcriptome of *G. lamblia* [29]. The general approach for identifying *G. lamblia* nucleolar proteins and reconstructing the *Gi*NuP is summarized in Fig. 5.

### Reconstruction of the ‘Higher Eukaryote Basic Nucleolar Proteome (HEBNuP)’

The orthologous relationships between any two of the three eukaryotes, *H. sapiens, A. thaliana,* and *S. cerevisiae*, were obtained from InParanoid database (http://inparanoid.sbc.su.se/cgi-bin/index.cgi) [46]. Orthologous nucleolar protein groups among all the three species were generated by MultiParanoid [47] based on the pairwise orthologous relationships. The IPI IDs of human nucleolar proteins were used to replace their corresponding Ensembl IDs in the orthologous groups through a local BLASTP search against the whole human proteome database in Ensembl with the 4057 human nucleolar proteins as queries (E-value cutoff 1e-10). The orthologous groups shared by human nucleolar proteome and the whole proteomes (in genome databases) of *Arabidopsis* and yeast were put together to reconstruct the ‘Higher Eukaryote Basic Nucleolar Proteome (HEBNuP)’.

### Functional inventory of the proteins in the *Gi*NuP and HEBNuP

The GO functional annotation of each human nucleolar protein was from the Gene Ontology database. Because no GO functional annotation of *G. lamblia* proteins is available to date, the GO functional annotations of *G. lamblia* nucleolar protein orthologs were classified according to the GO functional annotations of corresponding human nucleolar proteins in the same ortholog group. Ortholog groups among the *G. lamblia, H. sapiens, A. thaliana,* and *S. cerevisiae*, were generated by MultiParanoid as described above. Based on the identified nucleolar functions previously [1–5], we classified the nucleolar proteins into the following six main functional categories: 1) “ribosome related”, for example, ‘rRNA processing’; 2) “mRNA related”, for example, ‘mRNA processing’; 3) “translation related”, for example, ‘translation initiation factor’; 4) “DNA binding related”, for example, ‘DNA binding’; 5) “chromatin related”, for example, ‘chromatin remodeling complex’; and 6) “mitotic cell cycle related”, for example, ‘M/G1 transition of mitotic cell cycle’. Then the nucleolar proteins in *Gi*NuP and HEBNuP were inventoried by the six categories.

### Comparative analysis between *Gi*NuP and HEBNuP

Perl scripts were written to compare the *Gi*NuP with HEBNuP compositionally and functionally. Besides the *Gi*NuP dataset, four other datasets of nucleolar proteins were constructed: 1) HEBNuP-*Gi*NuP-shared dataset: the common proteins shared by both the *Gi*NuP and HEBNuP; 2) *Gi*NuP-specific dataset: the proteins being exclusively present in *Gi*NuP; 3) HEBNuP-specific dataset: the proteins being exclusively present in HEBNuP; 4) HEBNuP-*Hu* dataset: all the human nucleolar proteins in HEBNuP. Functional inventories of the proteins in all the five datasets were also carried out as above. Finally, comparisons of the six main and well-known nucleolar functional classes among the five datasets were implemented.

## Supplementary information

**Additional file 1: Supplementary Table S1.** The putative nucleolar proteome of *G. lamblia* (GiNuP) contains 255 individual nucleolar proteins.

**Additional file 2: Supplementary Table S2.** 799 orthologous groups among human nucleolar proteome, the whole proteome of *Arabidopsis*, and budding yeast proteome.

**Additional file 3: Supplementary Table S3.** The IDs and sequences of the nucleolar proteins of *Homo sapiens*, *A. thaliana*, and *S. cerevisiae* used in this work.

**Additional file 4: Supplementary Table S4.** Results of prediction of localization of *Giardia*-specific nucleolar proteins by the ProLoc program, the SubNucPred program, and the LOCTREE3 program.

## Data Availability

The whole human genome data was downloaded from Ensembl, and people can access to the recently updated data via the direct web link [ftp://ftp.ensembl.org/pub/release-99/fasta/homo_sapiens/pep/]. The *G. lamblia* genome data (GiardiaDB 3.1 released) was downloaded from the GiardiaDB via the link [https://giardiadb.org/giardiadb/showXmlDataContent.do?name=XmlQuestions.News#giardia05_13_news]. All data generated during this study are included within the paper and/or additional files.

## Abbreviations

AtNoPDB: Arabidopsis nucleolar protein database
BRH: Best Reciprocal Hit
*Gi*NuP: Giardial nucleolar proteome
GO: Gene Ontology
GPL: Glycerophospholipid
HEBNuP: Higher Eukaryote Basic Nucleolar Proteome
IPI: International Protein Index
NCBI: National Center for Biotechnology Information
NLS: Nuclear location signal
NORs: Nucleolar organizer regions
nr: non-redundant
rRNA: ribosome RNA

## References

[1] Boisvert FM, van Koningsbruggen S, Navascues J, Lamond AI (2007). The multifunctional nucleolus. Natl Rev.

[2] Feng JM, Sun J, Wen JF (2012). Advances in the study of the nucleolus. Zool Res.

[3] Larsen DH, Stucki M (2016). Nucleolar responses to DNA double-strand breaks. Nucleic Acids Res.

[4] Shaw P, Brown J (2011). Nucleoli: composition, function, and dynamics. Plant Physiol.

[5] Takada H, Kurisaki A (2015). Emerging roles of nucleolar and ribosomal proteins in cancer, development, and aging. Cell Mol Life Sci.

[6] Frottin F, Schueder F, Tiwary S, Gupta R, Korner R, Schlichthaerle T (2019). The nucleolus functions as a phase-separated protein quality control compartment. Science.

[7] Ahmad Y, Boisvert FM, Gregor P, Cobley A, Lamond AI (2009). NOPdb: nucleolar proteome database--2008 update. Nucleic Acids Res.

[8] Pendle AF, Clark GP, Boon R, Lewandowska D, Lam YW, Andersen J (2005). Proteomic analysis of the Arabidopsis nucleolus suggests novel nucleolar functions. Mol Biol Cell.

[9] Huh WK, Falvo JV, Gerke LC, Carroll AS, Howson RW, Weissman JS (2003). Global analysis of protein localization in budding yeast. Nature.

[10] Ogawa LM, Baserga SJ (2017). Crosstalk between the nucleolus and the DNA damage response. Mol BioSyst.

[11] Morrison HG, McArthur AG, Gillin FD, Aley SB, Adam RD, Olsen GJ (2007). Genomic minimalism in the early diverging intestinal parasite Giardia lamblia. Science.

[12] Gillin FD, Reiner DS, McCaffery JM (1996). Cell biology of the primitive eukaryote Giardia lamblia. Annu Rev Microbiol.

[13] Narcisi EM, Glover CV, Fechheimer M (1998). Fibrillarin, a conserved pre-ribosomal RNA processing protein of giardia. J Eukaryot Microbiol.

[14] Guo J, Chen YH, Zhou KY, Li JY (2005). Distribution of rDNA in the nucleus of Giardia lamblia - detection by ag-I silver stain. Anal Quant Cytol Histol.

[15] Sogin ML, Gunderson JH, Elwood HJ, Alonso RA, Peattie DA (1989). Phylogenetic meaning of the kingdom concept: an unusual ribosomal RNA from Giardia lamblia. Science.

[16] Cavalier-Smith T, Chao EE (1996). Molecular phylogeny of the free-living archezoan Trepomonas agilis and the nature of the first eukaryote. J Mol Evol.

[17] Hashimoto T, Nakamura Y, Kamaishi T, Nakamura F, Adachi J, Okamoto K (1995). Phylogenetic place of mitochondrion-lacking protozoan, Giardia lamblia, inferred from amino acid sequences of elongation factor 2. Mol Biol Evol.

[18] Hashimoto T, Nakamura Y, Nakamura F, Shirakura T, Adachi J, Goto N (1994). Protein phylogeny gives a robust estimation for early divergences of eukaryotes: phylogenetic place of a mitochondria-lacking protozoan, *Giardia lamblia*. Mol Biol Evol.

[19] Tovar J, Leon-Avila G, Sanchez LB, Sutak R, Tachezy J, van der Giezen M (2003). Mitochondrial remnant organelles of giardia function in iron-Sulphur protein maturation. Nature.

[20] Jimenez-Garcia LF, Zavala G, Chavez-Munguia B, Ramos-Godinez Mdel P, Lopez-Velazquez G, Segura-Valdez Mde L (2008). Identification of nucleoli in the early branching protist giardia duodenalis. Int J Parasitol.

[21] Hampl V, Hug L, Leigh JW, Dacks JB, Lang BF, Simpson AG (2009). Phylogenomic analyses support the monophyly of Excavata and resolve relationships among eukaryotic “supergroups”. Proc Natl Acad Sci U S A.

[22] Burki F (2014). The eukaryotic tree of life from a global phylogenomic perspective. Cold Spring Harb Perspect Biol.

[23] Lloyd D, Harris JC (2002). Giardia: highly evolved parasite or early branching eukaryote?. Trends Microbiol.

[24] Cernikova L, Faso C, Hehl AB (2018). Five facts about Giardia lamblia. PLoS Pathog.

[25] Nino CA, Chaparro J, Soffientini P, Polo S, Wasserman M (2013). Ubiquitination dynamics in the early-branching eukaryote Giardia intestinalis. MicrobiologyOpen.

[26] Gourguechon S, Holt LJ, Cande WZ (2013). The giardia cell cycle progresses independently of the anaphase-promoting complex. J Cell Sci.

[27] Feng JM, Sun J, Xin DD, Wen JF (2012). Comparative analysis of the 5S rRNA and its associated proteins reveals unique primitive rather than parasitic features in Giardia lamblia. PLoS One.

[28] Ye Q, Tian H, Chen B, Shao J, Qin Y, Wen J (2017). Giardia’s primitive GPL biosynthesis pathways with parasitic adaptation ‘patches’: implications for Giardia’s evolutionary history and for finding targets against giardiasis. Sci Rep.

[29] Franzen O, Jerlstrom-Hultqvist J, Einarsson E, Ankarklev J, Ferella M, Andersson B (2013). Transcriptome profiling of Giardia intestinalis using Strand-specific RNA-Seq. PLoS Comput Biol.

[30] Lara-Martinez R, De Lourdes S-VM, De La Mora-De La Mora I, Lopez-Velazquez G, Jimenez-Garcia LF (2016). Morphological studies of Nucleologenesis in Giardia lamblia. Anat Rec.

[31] Andersen JS, Lam YW, Leung AKL, Ong SE, Lyon CE, Lamond AI (2005). Nucleolar proteome dynamics. Nature.

[32] Xin DD, Wen JF (2005). Ribosome biogenesis system of giardia inferred from analysis of Giardial genome. Zool Res.

[33] Adam RD (2001). Biology of Giardia lamblia. Clin Microbiol Rev.

[34] Huala E, Dickerman AW, Garcia-Hernandez M, Weems D, Reiser L, LaFond F (2001). The Arabidopsis information resource (TAIR): a comprehensive database and web-based information retrieval, analysis, and visualization system for a model plant. Nucleic Acids Res.

[35] Rhee SY, Beavis W, Berardini TZ, Chen G, Dixon D, Doyle A (2003). The Arabidopsis information resource (TAIR): a model organism database providing a centralized, curated gateway to Arabidopsis biology, research materials and community. Nucleic Acids Res.

[36] Brown JW, Shaw PJ, Shaw P, Marshall DF (2005). Arabidopsis nucleolar protein database (AtNoPDB). Nucleic Acids Res.

[37] Cherry JM, Adler C, Ball C, Chervitz SA, Dwight SS, Hester ET (1998). SGD: saccharomyces genome database. Nucleic Acids Res.

[38] Christie KR, Weng S, Balakrishnan R, Costanzo MC, Dolinski K, Dwight SS (2004). Saccharomyces genome database (SGD) provides tools to identify and analyze sequences from Saccharomyces cerevisiae and related sequences from other organisms. Nucleic Acids Res.

[39] Hirschman JE, Balakrishnan R, Christie KR, Costanzo MC, Dwight SS, Engel SR (2006). Genome snapshot: a new resource at the saccharomyces genome database (SGD) presenting an overview of the Saccharomyces cerevisiae genome. Nucleic Acids Res.

[40] Guldener U, Munsterkotter M, Kastenmuller G, Strack N, van Helden J, Lemer C (2005). CYGD: the comprehensive yeast genome database. Nucleic Acids Res.

[41] Punta M, Coggill PC, Eberhardt RY, Mistry J, Tate J, Boursnell C (2012). The Pfam protein families database. Nucleic Acids Res.

[42] Horton P, Nakai K (1997). Better prediction of protein cellular localization sites with the k nearest neighbors classifier. Proc Int Conf Intell Syst Mol Biol.

[43] Huang WL, Tung CW, Huang HL, Hwang SF, Ho SY (2007). ProLoc: prediction of protein subnuclear localization using SVM with automatic selection from physicochemical composition features. Biosystems.

[44] Kumar R, Jain S, Kumari B, Kumar M (2014). Protein sub-nuclear localization prediction using SVM and Pfam domain information. PLoS One.

[45] Goldberg T, Hecht M, Hamp T, Karl T, Yachdav G, Ahmed N (2014). LocTree3 prediction of localization. Nucleic Acids Res.

[46] Remm M, Storm CEV, Sonnhammer ELL (2001). Automatic clustering of orthologs and in-paralogs from pairwise species comparisons. J Mol Biol.

[47] Alexeyenko A, Tamas I, Liu G, Sonnhammer ELL (2006). Automatic clustering of orthologs and inparalogs shared by multiple proteomes. Bioinformatics.

